# Assessing Clinical Embryology Research: A Global Bibliometric Analysis

**DOI:** 10.3390/medicina56050210

**Published:** 2020-04-26

**Authors:** Mara Simopoulou, Konstantinos Sfakianoudis, Evangelos Maziotis, Anna Rapani, Polina Giannelou, Agni Pantou, George Anifandis, Panagiotis Bakas, Nikolaos Vlahos, Konstantinos Pantos, Michael Koutsilieris

**Affiliations:** 1 Department of Physiology, Medical School, National and Kapodistrian University of Athens, 75, Mikras Asias, 11527 Athens, Greece; vagmaziotis@gmail.com (E.M.); rapanianna@gmail.com (A.R.); lina.giannelou@gmail.com (P.G.); mkoutsil@med.uoa.gr (M.K.); 2 Assisted Conception Unit, 2nd Department of Obstetrics and Gynecology, Aretaieion Hospital, Medical School, National and Kapodistrian University of Athens, 76, Vasilisis Sofias Avenue, 11528 Athens, Greece; p_bakas@yahoo.com (P.B.); nfvlahos@gmail.com (N.V.); 3 Centre for Human Reproduction, Genesis Athens Clinic, 14-16, Papanikoli, 15232 Athens, Greece; sfakianosc@yahoo.gr (K.S.); agnipantos@gmail.com (A.P.); info@pantos.gr (K.P.); 4 Department of Histology and Embryology, Faculty of Medicine, University of Thessaly, 3, Panepistimiou, 41500 Larisa, Greece; ganif@med.uth.gr

**Keywords:** bibliometrics, clinical embryology research, scientific production, publications, h-index

## Abstract

*Background and Objectives:* The evaluative strength of available bibliometric tools in the field of clinical embryology has never been examined in the literature. The aim is to bring insight regarding the identity of clinical embryology research, introducing concerns when solely relying on the methodology of bibliometric analysis. *Materials and Methods:* An all-inclusive analysis of the most bibliometrically highlighted scientific contributions regarding the cornerstones of clinical embryology was performed employing the Scopus, Web of Science (WoS) and PubMed databases, between 1978–2018. An analysis of the number of publications, respective citations and h-index, g-index, along with m-quotient is presented. The top 30 contributing authors for each distinctive area of research are listed. An attempt at visualizing the yearly published articles, clusters, and collaborations of authors, along with the geographic origin of publications, is also presented. *Results:* Combining all searches and keywords yielded 54,522 results. In the Scopus database, employing the keyword “In Vitro Fertilization” yielded 41,292 results. The publications of the top five authors in each research field were analytically presented and compared to the total number of publications for each respective field. The research field of Preimplantation Genetic Diagnosis/Screening/Testing was allocated the highest percentage of publications produced by the top five authors. Regarding journal bibliometrics, based on the year 2017 metrics, there are only 29 journals according to WoS that refer to “Reproductive Biology”, ranking it 187th among 235 disciplines. The USA produced the highest number of publications (12,537). *Conclusion:* Results indicate an explosion of interest published in the literature regarding the field of clinical embryology. Further analysis on collaborations and the trends involved should be of added value as productivity between countries varies significantly. This may guide researchers, in vitro fertilization professionals, and prospective authors during literature search, while proving useful regarding manuscript design and concurring on keywords and abstract content.

## 1. Introduction

Following the advent of assisted reproduction, clinical embryology has become an evolving field of interest in science. Presently, an enormous volume of scientific knowledge is freely available on clinical embryology. This reflects the novel approaches and ground-breaking research performed on the preimplantation embryo, toward an efficient and safe clinical practice in the era of precision medicine. However, the overwhelming number of published articles calls for an effective tool for managing the quantity and assessing the quality of the published academic research. The driver behind exploring the respective bibliographic databases could be fueled by various reasons and present with different levels of “search” for the practitioner of assisted reproduction. A terminal search could aim to fully capture a topic of research and advance knowledge of a specific field, as well as initiate or resume a debate regarding a current trend such as day-3 versus day-5 embryo transfer or optimal embryo biopsy timing for Preimplantation Genetic Diagnosis (PGD). It may focus on simply improving understanding of a subject or constitute the primary research work toward drafting a manuscript for publication. In any case, an effective search is essential and concurrently challenging to perform. The application of complimentary tools could potentially assist toward limiting, selecting, and deciding upon the degree of impact characterizing an article.

Perhaps the effort to depict academic distinction in a digestible way is challenging by nature as it is a multi-dimensional issue that fails to be properly addressed by simply applying mathematics and statistics. This fact renders this work timely and essential, uniquely bringing to the literature a first attempt to describing the body of clinical embryology in its entirety to date. This study aims to objectively and resourcefully portray the status of research in the field of clinical embryology by employing unbiased bibliometrics tools. The authors present the application of these tools in order to determine and define the currency all researchers should “pay” to establish their publication value in the competitive and fascinating world of science. Specifically, in the present study the authors (i) individually assess: “In vitro Fertilization”, “Intracytoplasmic Sperm Injection”, “cleavage stage embryo”, “blastocyst”, “embryo culture conditions”, “Preimplantation Genetic Diagnosis/Screening/Test” and “embryo cryopreservation”. (ii) present the most bibliometrically highlighted scientific contributions with regards to the milestones of clinical embryology, (iii) evaluate the difference in the number of publications longitudinally and geographically) present the top 30 contributing authors, for each distinctive area of research (iv) visualize the yearly published articles, clusters and collaborations of authors, along with the geographic origin of publications. Further to that, this article aims to assist the researcher and practitioner of assisted reproduction on how to better navigate the overwhelming volume of data published in the field of clinical embryology.

Bibliometrics consists of several mathematical and statistical tools applied to evaluate scholarly publications [1]. Theoretically, an influential published article will probably manage to affect other researchers’ future work, rendering that article’s value high. Certain indexes evaluate the influence an article exerts based on different criteria, although often the number of citations regarding a study is preferred [2]. Despite the variety of bibliometric tools that could be employed, none of them claims to be significantly superior to another, yet they remain a complimentary means if properly implemented during research.

Reporting on the number of articles published by an individual scientist is a simple way to perform a quantitative evaluation, yet not indicative of the value or quality of their work [3]. Besides the evaluation of authors, journals are ranked in order to indicate their productivity [2]. There are numerous author-level metrics including: total number of publications, total number of citations for all publications, average number of citations, author impact factor (IF) (summary of the IF of the journal that the author has produced publications in), h-index and g-index. H-index is a citation metric that mirrors the quantity and the quality of a specific author’s publications [4]. H represents the largest number of an author’s publications that were cited at least H times [5]. H- and g-indexes are similar, with the former being a preferred and more commonly employed option, though g-index is newer and mathematically more complex [6]. Nonetheless, h-index is still being considered to be a controversial author-level metric index. Another commonly employed index is the m-quotient (h-index/years) presenting the quality and quantity of the author’s publications in accordance to their publication years [7]. Finally, the h-real is the h-index of each author excluding self-citations.

In terms of journal-level metrics, IF provided by Web of Science (WoS) is the most accredited index. There are two other major indexes, called the Scimago Journal Ranking (SJR) and h5 index by Google Scholar. The field of “Reproductive Biology” according to WoS is named “Reproductive Medicine” in Scimago and “Reproductive Health” in Google Scholar.

## 2. Methods

### 2.1. Search Strategy

The WoS and Scopus database were thoroughly searched. Opting for these databases was a prerequisite since bibliometric tools are not available in other databases. The WoS was excluded due to returning duplicate publications and authors. Therefore, the Scopus database was used exclusively in order to limit the overlap in results for individual author searches. For example, a certain author may be searched and subsequently found in the results of the search with a different identity multiple times, depending on whether the search employed initials, the author’s first name, or surname. Moreover, when compared to WoS, the Scopus database offers 20% more coverage among citations in biomedical publications [8]. The search was performed in October 2018. Identified articles corresponded to publications from 1978-the year of the first publication on assisted reproduction-till 2018.Regarding journal data, the WoS database was used as it presents the most accredited IF (by Clarivate Analytics). The other indexes on journal data were extracted from Scimago and Google Scholar.

The keywords employed were “In vitro Fertilization”, “Intracytoplasmic Sperm Injection”, “cleavage stage embryo”, “blastocyst”, “embryo culture conditions”, “Preimplantation Genetic Diagnosis/Screening/Test” and “embryo cryopreservation”. These keywords were selected with the aim of fully representing not only the current and trending research areas in clinical embryology, but also aiming to portray the scientific interest in the field throughout decades of progress in clinical embryology. The selection was performed aiming toward an all-inclusive approach in presenting the field of clinical embryology. The rationale behind keywords’ selection was based on the observation that employing solely generalized search terms such as “In Vitro Fertilization” would result in failing to include a wide range of studies, among which some representing pivotal contributions in the field. On the other hand, excluding general keywords such as “In vitro Fertilization” would fail to yield some of the seminal older studies in the field.

The theoretical basis for deciding on inclusion and exclusion criteria was that our study exclusively referred to the practice of clinical embryology within the Assisted Reproductive Technology (ART). One limitation and obstacle in the data collection process was the inevitable inclusion of certain articles focusing on “animal models”, “developmental biology”, and “embryonic stem cells”, due to their relevance and high affinity to the field of clinical embryology. The keyword “animal models” could not be excluded as various studies on clinical embryology referred to animal model studies and included the specific phrase within the abstract. One striking example is the study by Edwards and Steptoe in 1978, which established the field of clinical embryology [9]. Similarly, the keyword “developmental” could also not be excluded since numerous studies refer to the developmental potential of the preimplantation embryo. The keyword “stem cells” was fully excluded from our study. Nonetheless, aiming to exclude articles from stem cell research could not account for articles on the same field describing them as pluripotent or totipotent. To overcome these identified limitations and exclude studies unrelated to the strict definition of clinical embryology practice, the subject area employed was limited to include only “Medicine”. The fields of “Veterinary”, “Agricultural and Biological Science”, “Neuroscience”, “Environmental Science”, “Engineering” and “Chemical Engineering” were excluded. Exclusion of these fields was decided in order to minimize the inclusion of possible non-human IVF studies. This search is fully reproducible and the exact search string is available in Supplementary Material A.

A similar search was performed on PubMed employing the “RISmed” package and analysis was performed employing the “bibliometrix” [10] package in R programming language. The aforementioned keywords were employed as MeSH terms. Since the “bibliometrix” package disregarded articles, it resulted in index score discrepancies. Therefore, author-level metrics obtained through this search were excluded, as they did not correspond to metrics provided by other databases. The authors with the highest number of publications were searched in Google Scholar database, employing “Publish or Perish” (6th edition) software [11] The network maps employed to present results of this analysis were created using the VOS viewer software (build 1.6.9). Retracted papers where obtained by retracted-watch database.

### 2.2. Statistical Analysis

The statistical analysis was performed in the R programming language. Differences in the number of publications were evaluated using the chi square test; similarity was assessed employing Jaccard’s index while rankings were evaluated employing Spearman’s Correlation coefficient. Confidence level was set at 95%.

## 3. Results

**Figure 1:**
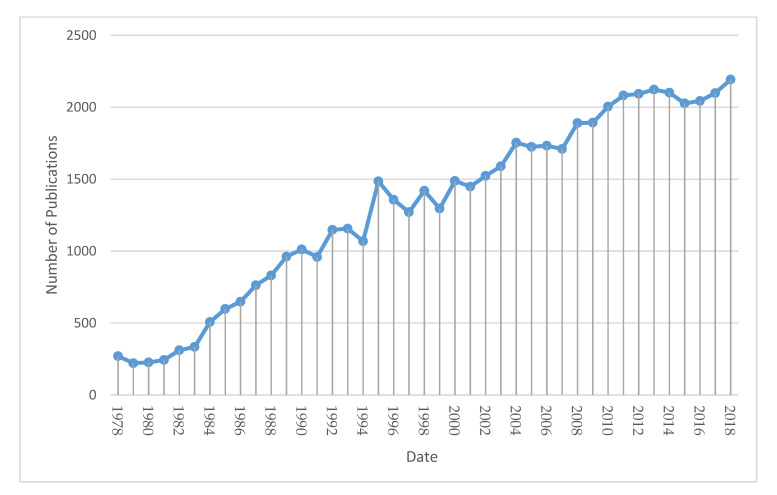
Graphical representation of published articles regarding the cumulative results of our search presented yearly, according to Scopus.

It should be highlighted that the field of clinical embryology is notably expanding, recently reaching almost 2100 publications per year, indicating an explosion of interest within the scientific community. The trend in publications presenting cumulative numbers of published scholarly articles evaluated yearly is presented in Figure 1.

### 3.1. Authors and Their Contributions

In the Scopus database, employing the keyword “In Vitro Fertilization” yielded 41,292 results. The author with the highest output was identified as Devroey P., having co-authored 384 publications. The keyword “ICSI” or Intracytoplasmic Sperm Injection” returned 8464 results. The author with the highest output was Devroey P., having co-authored 167 publications with the common denominator between them being the aforementioned keyword. The search string of the keyword “cleavage stage embryo” returned 2510 results, while the author with the highest output was identified to be Handyside A.H., with 27 publications in this area. The keyword “blastocyst” returned 9492 results. D.K. Gardner was the author with the highest output in the field with 83 publications. The same author also presented the highest output regarding “embryo culture conditions”, having co-authored 14 out of 1,981 publications in the specific field. In the field of “Preimplantation Genetic Diagnosis/Screening/Test” a total of 3778 publications are listed in the database, with Munné S. with the highest output of 123 publications. Finally, the search in the field of “embryo cryopreservation” returned 5770 results. The author presented the highest output was Devroey P., participating in 76 publications. The top 20 authors in each field are presented in Table S1.

The publications of the top five authors in each research field were analyzed and compared to the total number of publications in the respective field. The research field regarding Preimplantation Genetic Diagnosis/Screening/Test (PGD/PGT-A) contained highest percentage of publications produced by the top five authors (11%), followed by the search terms “ICSI” (7.3%), “embryo cryopreservation” (5%) and “cleavage stage embryo” (4.4%). The search terms “In Vitro Fertilization” and “blastocyst” presented with rates of 3.6% and 3.5% respectively. The search term with the lowest rate of publications conducted by the top five authors was embryo culture conditions with a rate of 2.4%.

When combining all of the above searches and keywords, a total of 54,522 results were generated. The author with the highest output was Devroey P. with 482. The search in PubMed yielded 64,075 results. The author with the highest output was Devroy P., similar to employing the Scopus search engine, albeit with a lower number of publications (447). A serious limitation of the search in PubMed was the number of articles for which the authors were not available (573). For the number of articles that correspond to “Reproductive Biology” the difference between PubMed and Scopus was statistically significant (*p* < 0.001).

**Figure 2:**
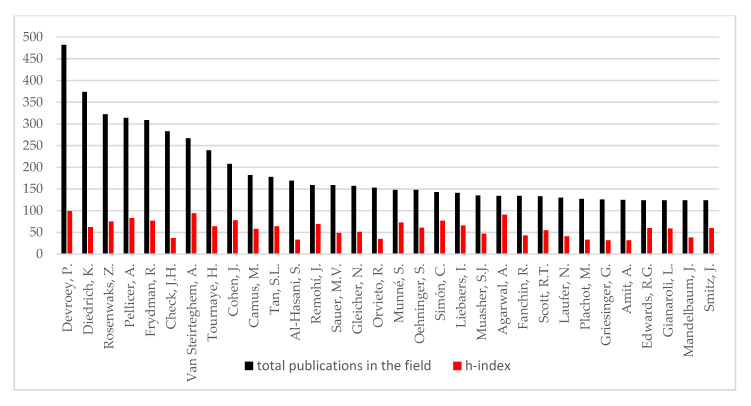
Number of publications in the field and h-index regarding the top 30 authors (order is defined by h-index).

**Figure 3:**
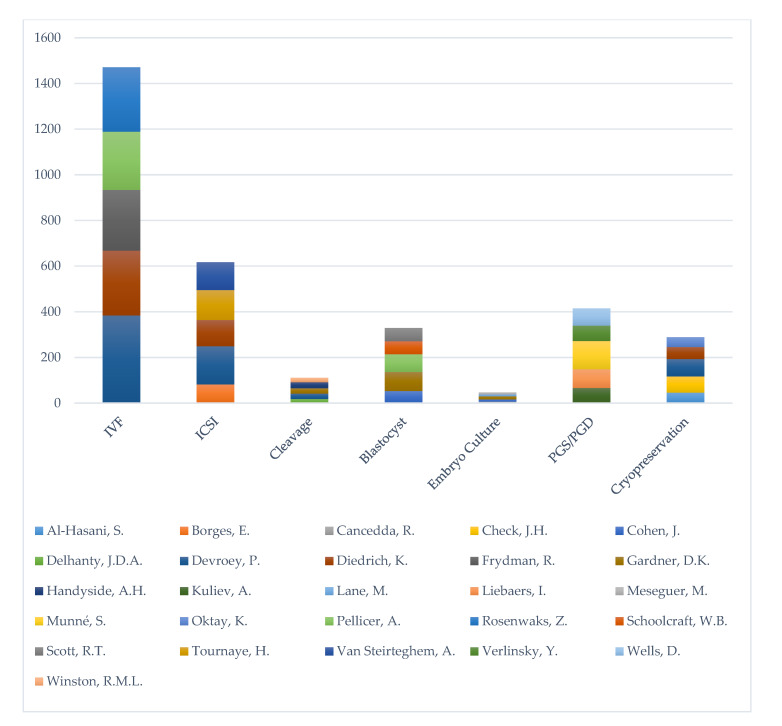
Number of publications for the top five authors in each sub-field in alphabetical order.

**Figure 4:**
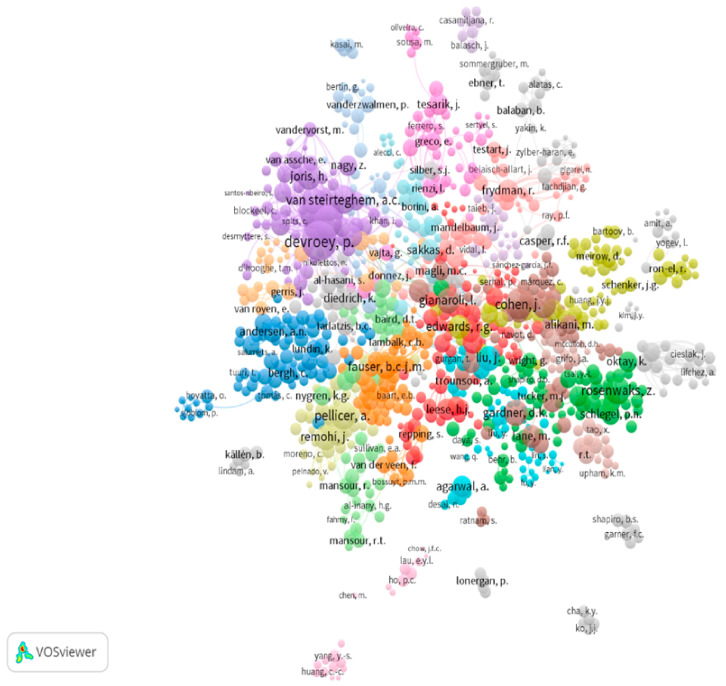
Clustering and network map of co-authorship. Magnitude of number of citations is indicated by font.

The h-index was evaluated for the top 30 authors in the clinical embryology field using Scopus. The h-index of the top five authors for each sub-field of the field was also evaluated. The h-index reported for the top five authors ranged from 100 for Devroey P. to 22 for Borges. The h-index of top authors for each sub-field is presented in Table 1 along with the number of publications and the number of documents that cited each author. The h-index and the number of publications in the field for the top 30 authors are presented in Figure 2, while the number of publications for the top five authors in each sub-field is presented in Figure 3. The h-index, g-index and m-quotient as obtained by both Google Scholar and Scopus are presented in Table 2 for the top 10 contributors. H-index -as provided by Scopus and Google Scholar- did not present any statistically significant difference. Figure 4 presents an attempt to chromatically visualize the clusters and collaborations of authors, while the magnitude of number of citations is highlighted and indicated by font size.

In an effort to present up-and-coming authors, an analysis of the publications and of the h-index of the top authors was performed for the five-year period from 2013–2018. The top 30 authors according to the assessed h-index based on the publications since 2013, are presented in Table 2. With the exception of five authors that are present in both Table 1 and Table 2, the remaining authors presented in Table 2 are in fact researchers that are emerging to be recognizably prolific in the field of clinical embryology. To evaluate the overlap between the two Tables, Jaccard’s index was used and was estimated at 0.098, meaning that the two tables share data and authors to an extent of 9.8%.

### 3.2. Analysis Per Country and Institution

**Figure 5:**
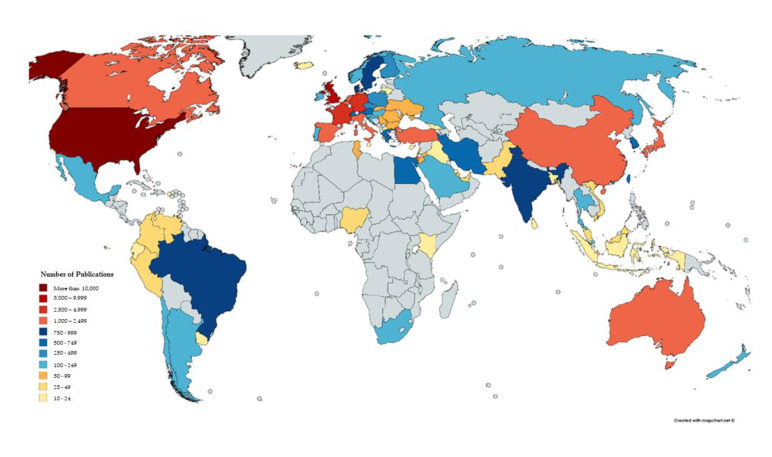
Publications by country (for countries with 10 or more publications), according to Scopus Database.

**Figure 6:**
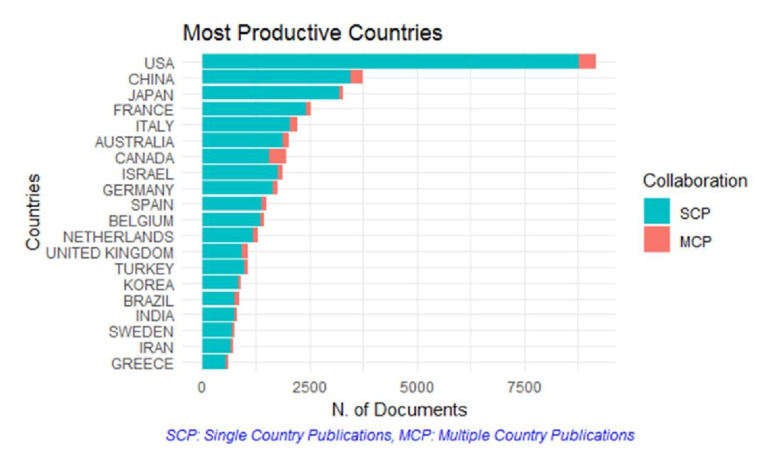
Single and multi-country publications according to PubMed results analysis.

The United States of America (USA) produced the highest number of publications (12,537), being more than double than those corresponding to the United Kingdom (UK) (5225) according to Scopus. The USA remained the country with the highest contribution when PubMed results were analyzed, with the second place being occupied by China, while the UK occupied the 13th place. Interestingly, according to Scopus, the most frequently encountered affiliation as reported by the authors was “Inserm”, the National Institute of France. The search in PubMed resulted in the Tel-Aviv University being the most frequent reported affiliation by authors. Nonetheless, the second most frequent reported affiliation was not available, rendering the results of the entire search as of low quality. Exact data for the top 20 countries and institutes are presented in Table 3. A world map depicting the publications by country -according to Scopus-is presented in Figure 5 and the ratio of single and multi-country publications -according to PubMed- is presented in Figure 6.

### 3.3. Journals

Regarding journal bibliometrics, based on the year 2017 metrics, there are only 29 journals according to WoS that refer to “Reproductive Biology”, ranking it 187th among 235 disciplines. Out of 29 journals, only four are not dedicated to human studies. According to Scimago, there are 70 journals reporting on “Reproductive Medicine”, and according to Google Scholar there are 20. The difference in number of journals in each list is statistically significant (*p* < 0.001). When ranked by the number of total citations number, “Reproductive Biology” ranks higher at 135th with more than 204,000 citations. Regarding the journal IF, the field of Reproductive Biology ranks 83rd on aggregate IF and 30th on the median IF category corresponding to the top 13th percentile. Only seven retracted articles were identified in the field of Reproductive Biology among the 54,522 articles (0.0128%). The comparison regarding the ranking of journals between the lists was evaluated using Spearman’s Correlation coefficient. In all three possible combinations the ranking was positively correlated, albeit not absolutely. Journal rankings according to each index are presented in Table 4.

### 3.4. Highly Cited Articles

When comparing on an article basis according to Scopus database, only four articles exceed 1000 citations and only one exceeds 2000 citations. The top-cited article in the field of clinical embryology is the “Pregnancies after intracytoplasmic injection of single spermatozoon into an oocyte”, the study that established the procedure of ICSI. Interestingly, the study that established the field, “Birth after the reimplantation of a human embryo”, is in second place. When comparing the mean citations per year “Pregnancies after intracytoplasmic injection of single spermatozoon into an oocyte” reaches again the top of the list with an average of 94.35 citations, followed by the 2005 “ESHRE guideline for the diagnosis and treatment of endometriosis” with an average of 72.54. The top-10 most cited articles are presented in Table 5.

## 4. Discussion

In the current study, we present an overall quantitative and qualitative assessment of authors’ performance in the field of clinical embryology, specifically regarding research on “In Vitro Fertilization”, “Intracytoplasmic Sperm Injection”, “cleavage stage embryo”, “blastocyst”, “embryo culture conditions”, “Preimplantation Genetic Diagnosis/Screening/Test”, as well as “embryo cryopreservation”. The respective areas of clinical embryology were researched and investigated separately. The h-index was used to contribute toward objectively reflect the scientific value of publications, their weight, and their identity within the volumes of studies available. Our results indicate that each field includes a diversity of unique authors who excel in their “arena of interest”. It should be further highlighted that the keyword yielding the highest number of publications was “blastocyst” followed by “ICSI”. This conclusion comes as no surprise since both “blastocyst” and “ICSI” have been in the spotlight of debate in the field of clinical embryology during recent decades describing the optimization of ART. The data regarding the top listed authors presented here may be viewed as portraying the foundation of research, as well as novel approaches in our field. The high percentage of publications produced by the top five authors (11%) was attributed to the research field of PGD/PGT-A reflecting the particular specialization of the field. Bibliometric tools succeeded in portraying and identifying authors that were expected to be yielded for each of the research areas investigated. Interestingly, the h-index attributed to the top listed authors’ ranks considerably high when compared to the list of the top h-index in the overall medical field, reflecting the value and prestige of the field of clinical embryology in medicine.

It should be noted that different databases yield different results. The search in Scopus and PubΜed resulted in a statistically significant difference in the number of articles. PubΜed fails as a means for bibliometric evaluation, as the only tool developed so far enabling bibliometric analysis in PubΜed provides a limited number of results. A possible reason behind this is the fact that PubΜed was not designed for bibliometric research, thus it requires huge computing power and numerous burdens in acquiring all the necessary data. It may be possible that modifications in the packages employed for mining citations from the PubMed database may enable bibliometric analysis in the future. It is worth mentioning that the “bibliometrix” package that was employed in this study, has only been available since last year, thus future upgrades may present as a solution to the aforementioned problems.

The journals on the field of Reproductive Biology are limited when compared to other disciplines. The high number of citations in the field, as well as the high ranking on journal IF metrics reflect the high quality of journals in the field. When evaluating from the perspective of the journals serving as the platform of communication of research, it is important to consider that the limited number of journals publishing on the field of clinical embryology correspond to a median IF that ranks high and reaches the top 13^th^ percentile as stated in our results. This fact is supported by the small number of retracted articles within this field proving the efficiency of the review process in place. The average retraction rate of 0.025% [12] is double compared to the one reported for the reproductive biology field. It should be mentioned that various different journal-level metrics exist. The most recognizable of these are IF by WoS, SJR by Scimago and h5-index by Google Scholar. The three different indexes provide alternative scoring systems regarding journal metrics. Although the parameters assessed for scoring are different, the ranking is positively correlated between all indexes indicating a high level of agreement.

**Figure 7:**
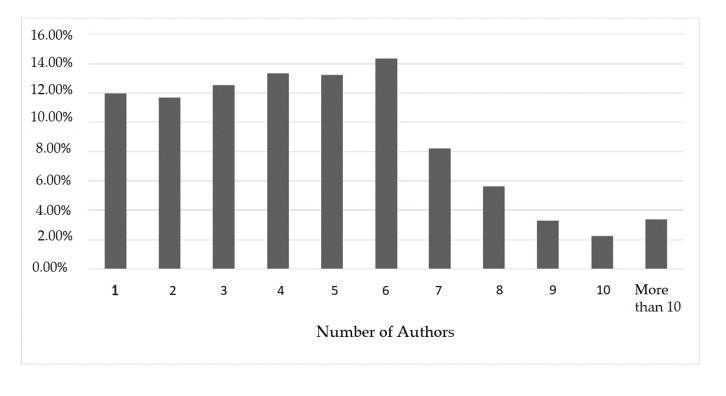
Percentage of articles according to the number of authors that contributed in producing them.

Highlighting the authors and respective research groups performing advanced research in several fields of clinical embryology was a principal aim of our investigation, as well as assessing their respective contribution among highly valued publications. However, it should be noted that these authors primarily present data sourced from team effort and therefore the respective articles do not correspond strictly to an individual researcher’s contribution. Each author’s contribution within a manuscript may vary, since different tasks may be executed by different authors in most published articles. Notably, the most frequent number of authors in each paper is 6, though there was a decline in the number of articles listing seven or more authors. A visual representation of the number of authors per publication is presented in Figure 7. Although examining publications under the prism of team performance may be more appropriate, it is extremely challenging to approach effectively. This is attributed to the fact that research groups tend to vary and are subject to change, with the exception of the senior author also known as “last author” serving as the leader, or senior supervisor of the research group. Nonetheless, the concept of team contribution was qualitatively represented in this study as the network map seen in Figure 4, which shows the co-authors that tend to publish collaboratively. Approaching research productivity according to geographical classifications is of interest. As seen in Figure 5 and Figure 6 the majority of publications originate from North America followed by East Asia and European countries, as expected with regards to investment in research. It was documented that international collaborations are found to be more common in top productive countries. As productivity between countries varies significantly analyzing collaborations may be of benefit, serving as a driver to promote research. The degree and nature of collaborations along with the topics would dictate advancement of the field. With respect to the analysis of the top authors corresponding to the last five years, it becomes apparent that there is a considerable influx of new researchers contributing significantly to the field, a fact confirming the ever evolving nature of clinical embryology. The Jaccard’s index set at 0.098 leads to the quantifiable conclusion that the majority of research publications from the past five years were contributed by “new” authors. The term “new” refers to researchers in clinical embryology that were not identified as top authors with regards to the past 40 years as presented in Table 1. In fact, considering that the overlap between Table 1 and Table 2 refers to five authors, it is of interest that this aforementioned majority of new contributors represents the 85.3% of the top authors of the last five years.

Bibliometrics present not only as a useful tool in the hands of the person conducting a research, but also for the scientist acting as an author. Evaluating the impact of a published article is a requisite for all authors who wish to assess the reflection of their work in the scientific community [2]. However, unprincipled prerequisites should be considered such as the precondition of citing specific journals in an article before an author is allowed to publish there [13]. Moreover, the concept of negative citations is interesting, as instances are recorded when articles are not cited on the grounds of supporting or disagreeing with the respective conclusions, but rather to dispute the statements presented therein [14]. Perhaps accounting for negative citations could be addressed in future analyses.

As anticipated, the papers that introduced and established the field and some of the sub-fields of clinical embryology ranked at the top of the list regarding the number of citations. Most top-cited studies were published in the 00′s (4 studies), followed by the 90′s (3 studies), the 80′s (2 studies) and the 70′s (1 study). Among the top-10, three papers served to introduce and establish the respective fields, two papers present guidelines and two others are systematic reviews and/or meta-analyses.

**Figure 8:**
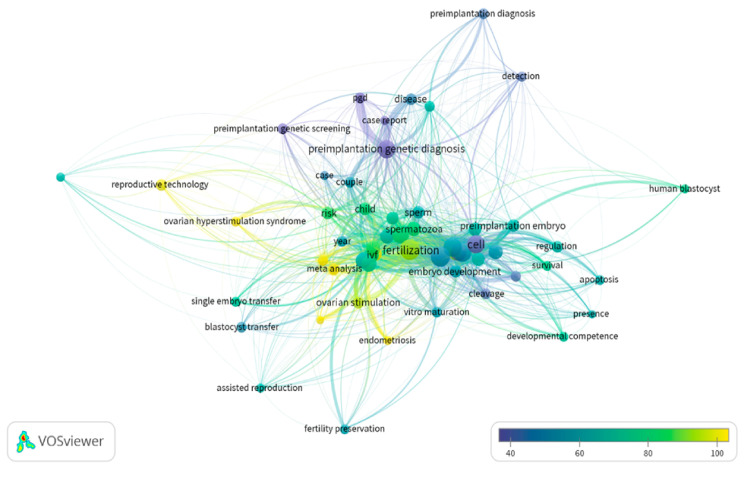
Network map and clustering on keywords as they were detected in abstracts.

**Figure 9:**
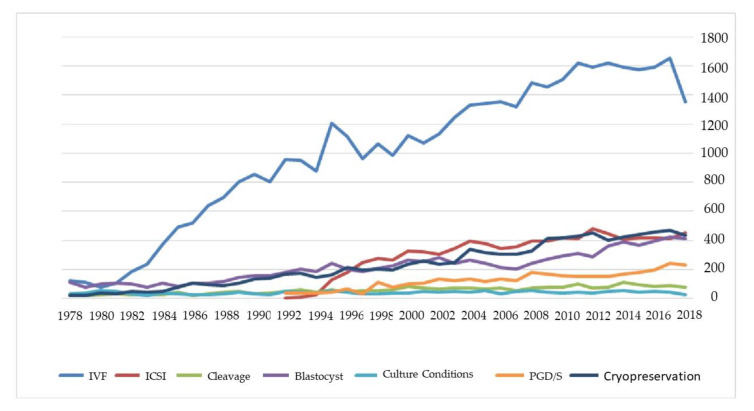
Number of articles on each keyword on the field of clinical embryology during the years.

This article attempts to raise authors’ awareness regarding several essential issues arising from this study. The selection of keywords, the suitability of the title and an accurate abstract section are matters that merit further attention prior to publishing an article. While conducting our investigation, there was no overlap as one would expect to encounter, between results yielded from the keywords used. The keywords “In Vitro Fertilization” and “Assisted Reproduction” yielded articles that did not overlap as expected, rendering the selection of appropriate keywords for our investigation a challenging task. Thus, when considering keywords prior to publication, it is important to ensure that one’s appropriately portrays the research involved as well as effectively communicates this to a targeted audience. The authors presented a network map and clustering on keywords as they were detected in abstracts. As shown in Figure 8 the word “fertilization” appears to be common among all possible searches, whereas “blastocyst” and “preimplantation diagnosis” appear to be more secluded. Nonetheless, it is clear that synonyms or interchangeable terms may vary in increasing or diminishing an article’s index value. Figure 9 shows the number of articles employing these keywords throughout the years, portraying the extent to which they reflect the strength of the various research sub-fields. Throughout the years the term “In Vitro Fertilization” appears to be the most commonly used in studies regarding reproductive biology and clinical embryology. It should be emphasized that the topics of “PGD/PGT-A” and “ICSI” were more recently introduced to the field when compared to “In Vitro Fertilization” or “culture media”.

Inevitably this bibliometric analysis included studies that may not be strictly related to clinical embryology as it is practiced in the context of ART. Several studies may be related to developmental biology or employing animal models. These limitations are explained in detail in the materials and methods section pertaining to inclusion exclusion criteria and respective decision-making. Moreover, since this study is a bibliometric analysis it “carries” the limitations of the databases employed. The WoS and Scopus databases require subscription-both from the readership and the journals-thus several studies published in journals not indexed in the above databases could not be included. Each citation tracking database presented with specific weaknesses. In the Scopus database citations to pre-1996 articles from post-1996 articles are not included in the authors h-index calculation [15]. The WoS database covers a narrower range of journals [16]. The Google Scholar database includes several non-peer-reviewed sources (i.e., dissertations) [17].

The process of researching published articles of interest may focus on researching names of praiseworthy authors and testing key words, despite the fact that at times they both fail to accomplish the desired end-point. The data presented may assist researchers toward navigating the overwhelming volume of publications, toward filtering and evaluating published articles in the era of precision medicine. It is common knowledge to experienced embryologists and practitioners that clinical embryology is evolving fast. Nonetheless, that may not apply to younger colleagues in the field who may find such information interesting and valuable. The holy grail of researching remains to be a less perplexing and time consuming, albeit more effective “search”. It appears that bibliometrics may present such a promise. Nonetheless, it should be noted that a tool employed to improve the system could result in more troubling consequences, such as defining an “elite” among research groups and respective first and senior authors. Furthermore, acquiring a list of prestigious authors may result in predisposed reviewers [4] and “intolerant” journals toward what may be described as “weaker” authors.

The authors refrain from suggesting means or a strategy to improve use of bibliometric tools, as they stand independently and that is the strength conveyed by mathematical concepts and approaches which represent the core of bibliometrics. It is our view that bibliometric tools should be employed within the scope of conducting research, familiarizing, and comprehending a certain topic in depth by identifying prized authors. In the era of abundance of information, research engines and overwhelming amounts of data, bibliometrics should be employed in making the decision to prioritize. However, the perils involved at solely relying on bibliometric tools should be highlighted. One could ponder, “how could an important scientific contribution by a new search group counterbalance its bibliometric weakness and successfully reach its target audience?” New research groups embarking on investigations may be underrepresented and hence fail to reach the wider audience with the same ease, a fact that should be accounted for. Whether bibliometric analysis is a valuable tool for evaluating performance is clear. Nonetheless, whether it is sufficient for bibliometric analysis alone to provide an overall evaluation is still unclear. Until a more complex formula is introduced—as suggested by Hicks [17]—reading and individually evaluating a published work is the most appropriate and robust way to assess an individual’s expertise. Mathematics and their objective nature may not always be the answer. The perception and personal judgment of each respective reader are fundamental components of the process of evaluation.

This is the first study to present an assessment of publications in the field of clinical embryology along with specific sub-fields. This study attempts to familiarize scientists within the field of clinical embryology with bibliometric markers. Recruiting bibliometric tools may in turn enable an all-inclusive evaluation of the clinical embryology research field as a whole, or a thorough presentation of the individualized value of a single article contribution. Interestingly, clinical embryology is a field where novel approaches are frequently introduced and investigated prior to establishing clinical routine practice status. In light of that fact, published data pertaining to the application of such novel approaches should be carefully evaluated further taking into account the respective bibliometric profile. It is widely accepted that certain types of studies weigh in with a higher cofactor in the equation of an article’s importance. This is heightened especially when such studies make statements with regards to optimal clinical practice. Nonetheless, bibliometric evaluation does not reflect on whether the article presents a triple blind randomized control study or a retrospective observational study, a longitudinal study or a case report, a meta-analysis, or a narrative review. Bibliometric analysis may further assist in identifying the “stronger” contributions with respect to reaching a larger audience and making an impact in future studies as shown by citation analysis. The focus of this work was not to discuss the performance of top listed authors. This is clearly presented in the results section and no further analysis is required. Their contribution to clinical embryology was unanimously recognized on various levels, since it undoubtably progressed the field and conveyed excellence in guiding safe and effective practice. Results sourced herein may serve to guide researchers on future studies. Taking into account bibliometric analysis when identifying a topic or a hypothesis worth exploring-being a novel concept or one that fuels a heated debate even-is of value. Further to that bibliometric analysis may even contribute in decision-making regarding future studies from study design, to selecting a title for an article as well as appropriate key words. However, the fact that time-sometimes extensive-is required for a published paper to be fully bibliometrically evaluated should be highlighted. Evaluating the impact that a scientific team’s work exerts through the citations corresponding to a publication may entail confounders. Recent publications may remain bibliometrically “concealed” with respect to citations that will subsequently profile the h-index for the respective authors. The period required for an article to be bibliometrically recognized is defined by the period that is required for drafting, submitting, revising, and publishing future relevant articles that will include it in their reference list.

## 5. Conclusions

This article provides an all-inclusive analysis employing bibliometric tools to assess the level of clinical embryology research. The authors discuss top-ranked contributions, countries, and journals while considerations and special considerations raised are indicated. When called to assess the overall performance of the field employing bibliometric tools it should be highlighted that clinical embryology was introduced in the literature in 1978 and it is, therefore, considered a rather “young” research field in Medicine. This fact may set the tone accordingly. Notably, a steady increase in scientific interest and production was observed in the field of clinical embryology since its conception marking the continuous improvements that are respectively documented in clinical practice.

## Figures and Tables

**Table 1:** Number of publications in the field, h-index, publications and number of citations for each author.

Field	Author Name	Publications in the Field	h-Index	Total Publications	Cited by Documents
In Vitro Fertilization (IVF)	Devroey, P.	384	99	673	19,655
	Diedrich, K.	284	62	1032	11,997
	Rosenwaks, Z.	282	75	511	15,463
	Frydman, R.	266	77	791	14,861
	Pellicer, A.	255	83	680	14,569
Intracytoplasmic Sperm Injection (ICSI)	Devroey, P.	167	99	673	19,655
	Tournaye, H.	131	64	404	9545
	Van Steirteghem, A.	122	94	583	18,349
	Diedrich, K.	115	62	1032	11,997
	Borges, E.	82	22	152	1374
Cleavage stage	Handyside, A.H.	27	55	175	11,526
	Devroey, P.	24	99	673	19,655
	Gardner, D.K.	23	71	202	7587
	Winston, R.M.L.	19	58	206	7312
	Delhanty, J.D.A.	18	44	208	4559
Blastocyst	Gardner, D.K.	83	71	202	7587
	Pellicer, A.	78	83	680	14,569
	Scott, R.T.	58	55	247	6466
	Schoolcraft, W.B.	57	43	125	5520
	Cohen, J.	53	78	317	9628
Pre-implantation Genetic Screening/Test/Diagnosis	Munné, S.	123	73	241	5771
	Liebaers, I.	83	66	307	9331
	Wells, D.	75	49	165	3968
	Verlinsky, Y.	68	43	150	3520
	Kuliev, A.	66	41	149	2772
Embryo culture conditions	Gardner, D.K.	14	71	202	7587
	Cohen, J.	9	78	317	9628
	Meseguer, M.	9	43	157	3220
	Lane, M.	8	58	159	6363
	Cancedda, R.	7	69	333	13,648
Embryo Cryopreservation	Devroey, P.	76	99	673	19,655
	Check, J.H.	71	37	368	3608
	Diedrich, K.	53	62	1032	11,997
	Al-Hasani, S.	46	33	246	3236
	Oktay, K.	43	52	195	5264

**Table 2:** The authors with h-index higher than 15 according to their publications since 2013.

Authors Name	Publications	h-Index
Mol, B.W.J.	43	37
Strowitzki, T.	29	34
Jamieson, D.J.	29	32
Pellicer, A.	38	26
Tournaye, H.	62	25
Pinborg, A.	32	25
Missmer, S.A.	32	24
Qiao, J.	59	23
Scott, R.T.	54	22
Kissin, D.M.	47	22
Somigliana, E.	44	22
Andersen, C.Y.	26	22
Rosenwaks, Z.	54	21
De Sutter, P.	38	21
Boulet, S.L.	34	20
Humaidan, P.	33	20
Rienzi, L.	32	20
Treff, N.R.	31	20
Chen, Z.J.	47	19
Gleicher, N.	43	19
Lambalk, C.B.	35	19
Polyzos, N.P.	34	19
Meseguer, M.	32	18
Repping, S.	31	18
Ubaldi, F.M.	30	18
Van Der Veen, F.	33	17
Orvieto, R.	45	16
Barad, D.H.	37	16
Li, R.	35	16
Jee, B.C.	34	15
Kushnir, V.A.	33	15
Verheyen, G.	32	15
Li, T.C.	28	15
Schoolcraft, W.B.	28	15

**Table 3:** Author-metrics indexes for the top 10 contributors as obtained by Scopus and Google Scholar.

Author	h-Index (Scholar)	h-Index (Scopus)	M Quotient (Scholar)	M Quotient (Scopus)	g-Index (Scholar)	Cites/Year (Scholar)	h-Real (Scopus)
Devroey P.	117	100	3.16	2.70	196	993	95
Diedrich K.	73	63	1.70	1.53	113	255	59
Rosenwanks Z.	93	75	2.26	1.83	153	305	73
Pellicer A.	95	83	2.43	2.13	145	115	80
Frydman R.	74	77	1.72	1.79	112	437	75
Check J.H.	42	37	1.02	0.90	60	161	26
Van Steirteghem A.	110	94	2.82	2.41	186	1015	91
Tournaye H.	70	64	2.5	2.29	114	554	62
Cohen J.	92	78	2.49	2.11	148	219	75
Camus M.	50	59	1.52	1.76	93	252	57

**Table 4:** Journal rankings according to different indexes.

	Impact Factor by WoS	H5-Index by Google Scholar	Journal Rankings by Scimago
Human Reproduction Update	11.852	74	5.317
Human Reproduction	4.99	71	2.643
Fertility And Sterility	4.803	83	2.25
Molecular Human Reproduction	3.449	40	1.619
Biology Of Reproduction	3.184	45	1.446
Molecular Reproduction And Development	3.113	26	1.134
Reproduction	3.086	42	1.322
Reproductive Biomedicine Online	2.967	47	1.343
Reproductive Biology and Endocrinology	2.852	38	1.203
Journal of Assisted Reproduction and Genetics	2.788	38	1.179
American Journal of Reproductive Immunology	2.745	38	1.21
Seminars in Reproductive Medicine	2.67	29	1.143
Reproductive Toxicology	2.58	38	0.846
Reproductive Sciences	2.548	36	1.001
Placenta	2.434	41	1.223
Journal of Ovarian Research	2.367	33	1.008
Journal of Reproductive Immunology	2.322	31	0.997
Reproduction Fertility and Development	2.105	24	0.681
European Journal of Obstetrics & Gynecology And Reproductive Biology	1.809	43	0.828
Journal of Reproduction And Development	1.635	21	0.725
Systems Biology in Reproductive Medicine	1.582	17	0.558
Reproductive Biology	1.446	19	0.668
Human Fertility	1.438	17	0.547
Zygote	1.11	16	0.387

**Table 5:** Top-Cited articles in the field.

Title	Authors	Year Published	Journal	Citations	Mean Citations Per Year
Pregnancies after intracytoplasmic injection of single spermatozoon into an oocyte	Palermo, G., Joris, H., Devroey, P., Van Steirteghem, A.C.	1992	The Lancet	2453	94.35
Birth after the reimplantation of a human embryo	Steptoe, P.C., Edwards, R.G.	1978	The Lancet	1330	33.25
Pregnancies from biopsied human preimplantation embryos sexed by Y-specific DNA amplification	Handyside, A.H., Kontogianni, E.H., Hardy, K., Winston, R.M.L.	1990	Nature	1073	38.32
High fertilization and implantation rates after intracytoplasmic sperm injection	Van Steirteghem, A.C., Nagy, Z., Joris, H., Liu, J., Staessen, C., Smitz, J., Wisanto, A., Devroey, P.	1993	Human Reproduction	1039	41.56
Sperm morphologic features as a prognostic factor in in vitro fertilization	Kruger, T.F., Menkveld, R., Stander, F.S.H., Lombard, C.J., Van der Merwe, J.P., van Zyl, J.A., Smith, K.	1986	Fertility and Sterility	959	29.96
ESHRE guideline for the diagnosis and treatment of endometriosis	Kennedy, S., Bergqvist, A., Chapron, C., D’Hooghe, T., Dunselman, G., Greb, R., Hummelshoj, L., Prentice, A., Saridogan, E., Koninckx, P., Matorras, R., Mueller, M., Garcia-Velasco, J.	2005	Human Reproduction	943	72.54
Human gene expression first occurs between the four- and eight-cell stages of preimplantation development	Braude, P., Bolton, V., Moore, S.	1988	Nature	928	30.93
Role of reactive oxygen species in the pathophysiology of human reproduction	Agarwal, A., Saleh, R.A., Bedaiwy, M.A.	2003	Fertility and Sterility	813	54.2
Perinatal outcomes in singletons following in vitro fertilization: A meta-analysis	Jackson, R.A., Gibson, K.A., Wu, Y.W., Croughan, M.S.	2004	Obstetrics and Gynecology	765	54.64
A systematic review of tests predicting ovarian reserve and IVF outcome	Broekmans, F.J., Kwee, J., Hendriks, D.J., Mol, B.W., Lambalk, C.B.	2006	Human Reproduction Update	759	63.25

## Supplementary Materials

The following are available online at https://www.mdpi.com/1010-660X/56/5/210/s1, Supplementary material A: Search strategy, Table S1: Top-20 authors in each field.
