# Supplementary material for: Assessing Clinical Embryology Research: A Global Bibliometric Analysis
Source: Medicina (Kaunas). 2020 Apr 26;56(5):210. doi: 10.3390/medicina56050210 (PMC7279470; doi:10.3390/medicina56050210)
Supplement: Supplementary file 1 [file medicina-56-00210-s001.pdf]

Supplementary material A.

1. (TITLE-ABS-KEY(In Vitro Fertilization) AND NOT (stem cells) AND (LIMIT-TO (SUBJAREA,"MEDI") OR EXCLUDE (SUBJAREA,"AGRI") OR EXCLUDE (SUBJAREA,"ENGI") OR EXCLUDE (SUBJAREA,"ENVI") OR EXCLUDE (SUBJAREA,"NEUR") OR EXCLUDE (SUBJAREA,"CENG") OR EXCLUDE (SUBJAREA,"VETE")))
2. (TITLE-ABS-KEY(ICSI OR (Intracellular Sperm Injection)) AND NOT (stem cells) AND (LIMIT-TO (SUBJAREA,"MEDI") OR EXCLUDE (SUBJAREA,"AGRI") OR EXCLUDE (SUBJAREA,"ENGI") OR EXCLUDE (SUBJAREA,"ENVI") OR EXCLUDE (SUBJAREA,"NEUR") OR EXCLUDE (SUBJAREA,"CENG") OR EXCLUDE (SUBJAREA,"VETE")))
3. (TITLE-ABS-KEY(cleavage stage embryo) AND NOT (stem cells) AND (LIMIT-TO (SUBJAREA,"MEDI") OR EXCLUDE (SUBJAREA,"AGRI") OR EXCLUDE (SUBJAREA,"ENGI") OR EXCLUDE (SUBJAREA,"ENVI") OR EXCLUDE (SUBJAREA,"NEUR") OR EXCLUDE (SUBJAREA,"CENG") OR EXCLUDE (SUBJAREA,"VETE")))
4. (TITLE-ABS-KEY(blastocyst) AND NOT (stem cells) AND (LIMIT-TO (SUBJAREA,"MEDI") OR EXCLUDE (SUBJAREA,"AGRI") OR EXCLUDE (SUBJAREA,"ENGI") OR EXCLUDE (SUBJAREA,"ENVI") OR EXCLUDE (SUBJAREA,"NEUR") OR EXCLUDE (SUBJAREA,"CENG") OR EXCLUDE (SUBJAREA,"VETE")))
5. (TITLE-ABS-KEY(Preimplantation AND Genetic AND (Screening OR Test OR Diagnosis)) AND NOT (stem cells) AND (LIMIT-TO (SUBJAREA,"MEDI") OR EXCLUDE (SUBJAREA,"AGRI") OR EXCLUDE (SUBJAREA,"ENGI") OR EXCLUDE (SUBJAREA,"ENVI") OR EXCLUDE (SUBJAREA,"NEUR") OR EXCLUDE (SUBJAREA,"CENG") OR EXCLUDE (SUBJAREA,"VETE")))

6. (TITLE-ABS-KEY(embryo AND culture AND conditions) AND NOT (stem cells) AND (LIMIT-TO (SUBJAREA,"MEDI") OR EXCLUDE (SUBJAREA,"AGRI") OR EXCLUDE (SUBJAREA,"ENGI") OR EXCLUDE (SUBJAREA,"ENVI") OR EXCLUDE (SUBJAREA,"NEUR")))
7. (TITLE-ABS-KEY((embryo OR oocyte OR sperm) AND (cryopreservation OR freezing OR thawing)) AND NOT (stem cells) AND (LIMIT-TO (SUBJAREA,"MEDI") OR EXCLUDE (SUBJAREA,"AGRI") OR EXCLUDE (SUBJAREA,"ENGI") OR EXCLUDE (SUBJAREA,"ENVI") OR EXCLUDE (SUBJAREA,"NEUR") OR EXCLUDE (SUBJAREA,"CENG") OR EXCLUDE (SUBJAREA,"VETE")))
8. #1 OR #2 OR #3 OR #4 OR #5 OR #6 OR #7

PubMed Search:

1. Fertilization, in Vitro
2. Sperm injection, Intracytoplasmic
3. Cleavage stage embryo
4. Blastocyst
5. Preimplantation Genetic AND (Diagnosis OR Screening OR Test)
6. Embryo culture conditions
7. (embryo OR oocyte OR sperm) AND (cryopreservation OR freezing OR thawing)
8. #1 OR #2 OR #3 OR #4 OR #5 OR #6 OR #7

Table S1: Top-20 authors in each field.

In-Vitro Fertilization	ICSI	Cleavage stage embryo	Blastocyst	Embryo culture conditions	PGD/PGS	Embryo Cryopre-servation
Devroey, P.	Devroey, P.	Handyside, A.H.	Gardner, D.K.	Munné, S.	Gardner, D.K.	Devroey, P.

Diedrich, K.	Tournaye, H.	Devroey, P.	Pellicer, A.	Liebaers, I.	Cohen, J.	Check, J.H.
Rosenwaks, Z.	Van Steirteghem, A.	Gardner, D.K.	Scott, R.T.	Wells, D.	Meseguer, M.	Diedrich, K.
Frydman, R.	Diedrich, K.	Winston, R.M.L.	Schoolcraft, W.B.	Verlinsky, Y.	Lane, M.	Al-Hasani, S.
Pellicer, A.	Borges, E.	Delhanty, J.D.A.	Cohen, J.	Kuliev, A.	Cancedda, R.	Oktay, K.
Check, J.H.	Al-Hasani, S.	Racowsky, C.	Simón, C.	Sermon, K.	Grosse, G.	Camus, M.
Van Steirteghem, A.	Iaconelli, A.	Cohen, J.	Remohí, J.	Handyside, A.H.	Leese, H.J.	Cohen, J.
Tan, S.L.	De Sutter, P.	Munné, S.	Leese, H.J.	Cohen, J.	Menezo, Y.	Tournaye, H.
Tournaye, H.	Liebaers, I.	Balaban, B.	Meseguer, M.	Devroey, P.	Pellicer, A.	Pellicer, A.
Cohen, J.	Verheyen, G.	Sakkas, D.	Devroey, P.	Van Steirteghem, A.	Thompson, J.G.	Rosenwaks, Z.
Camus, M.	Bonduelle, M.	Tournaye, H.	Sengupta, J.	Harper, J.C.	Tucker, M.J.	Frydman, R.
Gleicher, N.	Camus, M.	Harper, J.C.	Lane, M.	Scott, R.T.	Barrett, J.C.	Son, W.Y.
Sauer, M.V.	Palermo, G.D.	Rienzi, L.	Handyside, A.H.	Gianaroli, L.	Lindner, G.	Van Steirteghem, A.
Orvieto, R.	Pellicer, A.	Urman, B.	Wells, D.	Delhanty, J.D.A.	Rosenwaks, Z.	Chian, R.C.
Muasher, S.J.	Check, J.H.	Van Steirteghem, A.	Behr, B.	Staessen, C.	Sanford, K.K.	Mandelbaum, J.
Oehninger, S.	Kremer, J.A.M.	Wells, D.	Ghosh, D.	Treff, N.R.	Swain, J.E.	Tan, S.L.
Remohí, J.	Franco, J.G.	Tan, S.L.	Munné, S.	Ferraretti, A.P.	Barry, W.H.	Bergh, C.
Laufer, N.	Rosenwaks, Z.	Verheyen, G.	Murphy, C.R.	Traeger-Synodinos, J.	Bongso, A.	Choe, J.K.
Amit, A.	Dhont, M.	Ziebe, S.	Racowsky, C.	Diedrich, K.	Gilchrist, R.B.	Cobo, A.
Plachot, M.	Schlegel, P.N.	Ao, A.	Vanderzwalmen, P.	De Rycke, M.	Hardy, K.	Gianaroli, L.
